# Retraction Note: Melatonin enhances TNF-α-mediated cervical cancer HeLa cells death via suppressing CaMKII/Parkin/mitophagy axis

**DOI:** 10.1186/s12935-023-02922-w

**Published:** 2023-04-20

**Authors:** Qinghe Zhao, Wuliang Wang, Jinquan Cui

**Affiliations:** Department of Obstetrics and Gynecology, The Second Affiliated Hospital of Zhengzhou University, No.2 Jinba Road, Zhengzhou, 450014 China

**Retraction Note: Cancer Cell Int (2019) 19:58**

10.1186/s12935-019-0777-2

The Editors-in-Chief have retracted this article. After publication, concerns were raised regarding the data in several figures. Specifically:

- Figure 2a has very unusual shapes for flow cytometry plots.
- The mito-LC3 western blot in Fig. 4f appears highly similar to Fig. 3e CIII-core2 in [1], which was submitted and published within a similar time frame.
- There appears to be a vertical break in the p62 western blot background in Fig. 4f.

The authors have stated that these experiments were outsourced to a third party, and the raw data are no longer available. The Editors-in-Chief therefore no longer have confidence in the presented data.

All authors agree to this retraction.

## References

[1] Yao W, Zhu S, Li P (2019). Large tumor suppressor kinase 2 overexpression attenuates 5-FU-resistance in colorectal cancer via activating the JNK-MIEF1-mitochondrial division pathway. Cancer Cell Int.

